# Exploiting Visibility Information in Surface Reconstruction to Preserve Weakly Supported Surfaces

**DOI:** 10.1155/2014/798595

**Published:** 2014-08-11

**Authors:** Michal Jancosek, Tomas Pajdla

**Affiliations:** Centre for Machine Perception, Department of Cybernetics, Faculty of Electrical Engineering, Czech Technical University in Prague, Prague 166 27, Czech Republic

## Abstract

We present a novel method for 3D surface reconstruction from an input cloud of 3D points augmented with visibility information. We observe that it is possible to reconstruct surfaces that do not contain input points. Instead of modeling the surface from input points, we model free space from visibility information of the input points. The complement of the modeled free space is considered full space. The surface occurs at interface between the free and the full space. We show that under certain conditions a part of the full space surrounded by the free space must contain a real object also when the real object does not contain any input points; that is, an occluder reveals itself through occlusion. Our key contribution is the proposal of a new interface classifier that can also detect the occluder interface just from the visibility of input points. We use the interface classifier to modify the state-of-the-art surface reconstruction method so that it gains the ability to reconstruct weakly supported surfaces. We evaluate proposed method on datasets augmented with different levels of noise, undersampling, and amount of outliers. We show that the proposed method outperforms other methods in accuracy and ability to reconstruct weakly supported surfaces.

## 1. Introduction

The problem of surface reconstruction from input 3D points is well studied and different solutions are widely used in many industries. However, producing complete reconstructions of outdoor and complicated scenes is still an open problem. The input points can be obtained using different techniques. Laser scanners and structured-light based systems along with a registration method are probably the most widely used as sources of input points. Recently, images processed with a structure-from-motion (SfM) method [1–4] and a multiview-stereo (MVS) method [5–14] have become another popular source of input points. The input points can almost always (with respect to the acquisition technique) be augmented with visibility information. By visibility information of an input point, we mean that 3D position(s) of sensor(s) that produce(s) the input point is/are known. We consider input points that are close to the real surface but do not represent the real surface as noise and input points that are far from the real surface as outliers.

Surfaces that do not have strong support in the input points but represent real surfaces in the scene, that is,* weakly supported surfaces*, are essential for achieving complete reconstructions. Such surfaces may be transparent, highly reflective, and lacking in texture or, as in the case of the ground planes, they can be occluded by moving objects such as people and cars. weakly supported surfaces are present especially when the input points were obtained from images processed by a SfM method and a MVS method. Frequent examples are the ground planes in tourist photo collections, which are often blurred, since the cameras are mostly focused on objects of interest (facades and landmarks) and on people above the ground. Additionally, input points can contain relatively large amounts of outliers. The density of outlier points is often similar or greater than the density of points of a weakly supported surface. In this paper we propose a novel method that can reconstruct these difficult weakly supported surfaces.

## 2. Related Work

Here we review previous work that is most relevant to the method proposed in this paper.

Silhouette based methods are mostly related to the idea of visual hull that was introduced by Laurentini in [15]. This technique relies on the ability to clearly separate the scene objects from the background on input images. These objects silhouettes are used to infer shapes. A more robust approach exploiting the same idea is proposed in [16]. This approach introduces occupancy grid concept. It infers where and how likely the matter is present in the scene from given silhouette cues. The method proposed in [17] obtains moving occluder silhouette information from video sequences of static calibrated cameras using a background subtraction technique. A Bayesian sensor fusion formulation is used to process all occlusion cues in the multiview sequence. The method proposed in [18] is similar to the method proposed in [17] but applies state-of-the-art natural image matting algorithms for multiview sequences to obtain occluder silhouette cues. The image matting information is later merged into a 3D coordinate and a composite model is created. All these methods rely on silhouette cues information and use it to reconstruct occluders in the scene. Our method builds on another principle and does not need any occluder silhouette information.

Local depth-map filtering methods [8, 13, 19–22] alternate depth-map computation from images with depth-map filtering in order to obtain complete and outlier-less depth maps. Some of them [8, 13, 19, 20] use a visibility-based filtering approach. Another [22] uses buddle adjustment minimization of the camera reprojection error to filter outliers and improve accuracy. The final filtered depth maps are later processed by a depth-map fusion method, usually the method proposed in [23], to produce the final surface 3D reconstruction. The depth-map filtering methods are rather conservative and tend to reconstruct only those surfaces that are strongly supported by the input data. Unlike our method the local depth-map filtering methods leave the space free where the support is weak.

Volumetric depth-map fusion methods have achieved great success in recent years. Depth maps often contain many outliers and are noisy or noncomplete, especially when computed from images. Some volumetric depth-map fusion methods [24–26] are designed to be robust against outliers and to produce complete, accurate, and visibility-consistent results. Some of them [23] are focused to produce smooth and high quality results. Another volumetric depth-map fusion method [27, 28] can produce high quality result fast; however, it requires many and relatively high-quality depth maps. Our method falls into the group of robust volumetric depth-map fusion methods.

Space carving introduced in [29] produces an approximate reconstruction called photo hull, which is, under very strict conditions, guaranteed to subsume all other photo-consistent reconstructions. The assumptions are so strict that the method is useless for reconstruction of outdoor scenes. It also does not reconstruct weakly supported surfaces well since all non-photo-consistent volume is carved out. Photo flux was recently introduced in [30]. It is closely related to photo hull but allows the recovery of finer shape details without oversmoothing while still handling noise robustly. An approach to multiview image based 3D reconstruction by statistically inversing the ray-tracing based image generation process was presented in [31]. The method proposed in [25] uses octree partitioning of 3D space and computes the minimal* s*-*t* cut of a graph derived from the octree in order to label each voxel as being inside or outside. The discontinuity cost used in [25] is based on photo consistency (or sensor depth map). All previously mentioned methods proposed in [25, 29–31] rely on 3D reconstruction of visible, solid, well-textured, and static scenes from images. The photo consistency of the scene surface plays an important role in all these methods while non-photo-consistent parts are usually not reconstructed. The methods do not pay any particular attention to occluders and weakly supported surfaces. Moreover, the methods use voxel-based volumetric representation of the space. Since the number of voxels depends on the resolution of the volume cubically, the computational and memory costs quickly reach machine limits. For this reason, the voxel-based volumetric methods are suited for reconstruction of small and compact objects only.

The methods proposed in [24, 32, 33] work with Delaunay tetrahedralization of the input points instead of voxel-based volumetric representation. The tetrahedral-based volumetric representation suites better for large real-world reconstructions than voxel-based one. Size of tetrahedra depends just on the spatial distribution and density of input points. Therefore we have adopted the tetrahedral volumetric representation in our method.

Pioneered by [24], both the method proposed in [33] and the method proposed in [24] are based on a minimal* s*-*t* cut approach. In both methods, an* s*-*t* graph is derived from the Delaunay tetrahedralization of the input points in order to reconstruct the final surface. The minimal* s*-*t* cut of the graph labels each tetrahedra as “being full” with a full label or “being free” with a free label. The final surface consists of the faces shared by the tetrahedra labeled as being “free” (empty space) and the tetrahedra labeled as being “full” (full space). Therefore, the surface can be seen as an* interface* between the free and the full spaces. The new element in the method proposed in [33] with respect to the method proposed in [24] is that it can reconstruct* weakly supported surfaces*. We provide a detailed comparison of both [24, 33] methods with respect to the newly proposed method later in the text.

*Contributions.* The method proposed in this work is a generalization of the method proposed in [33]. The main contribution of the newly proposed method is that it produces more accurate results than the method proposed in [33] and accordingly is able to reconstruct weakly supported surfaces better. We propose a new interface classifier and we justify the design of the interface classifier by experiments. We show experimentally that the number of false positives (wrongly classified non-*interface* points as* interface*) is negligible and that the number of true positives (especially on* weakly supported surfaces*) is considerable. We demonstrate the superior performance of the proposed method over the methods proposed in [23, 24, 33] on several different datasets and provide a quantitative evaluation.

## 3. Weakly Supported Surfaces Reveal Themselves through Occlusion

The main idea of improving the reconstruction of weakly supported surfaces in [33] is that even weakly supported surfaces exhibit themselves by occluding other input points. We next review this idea for an infinite number of noiseless and outlier-less input points augmented with visibility information.

**Figure 1:**
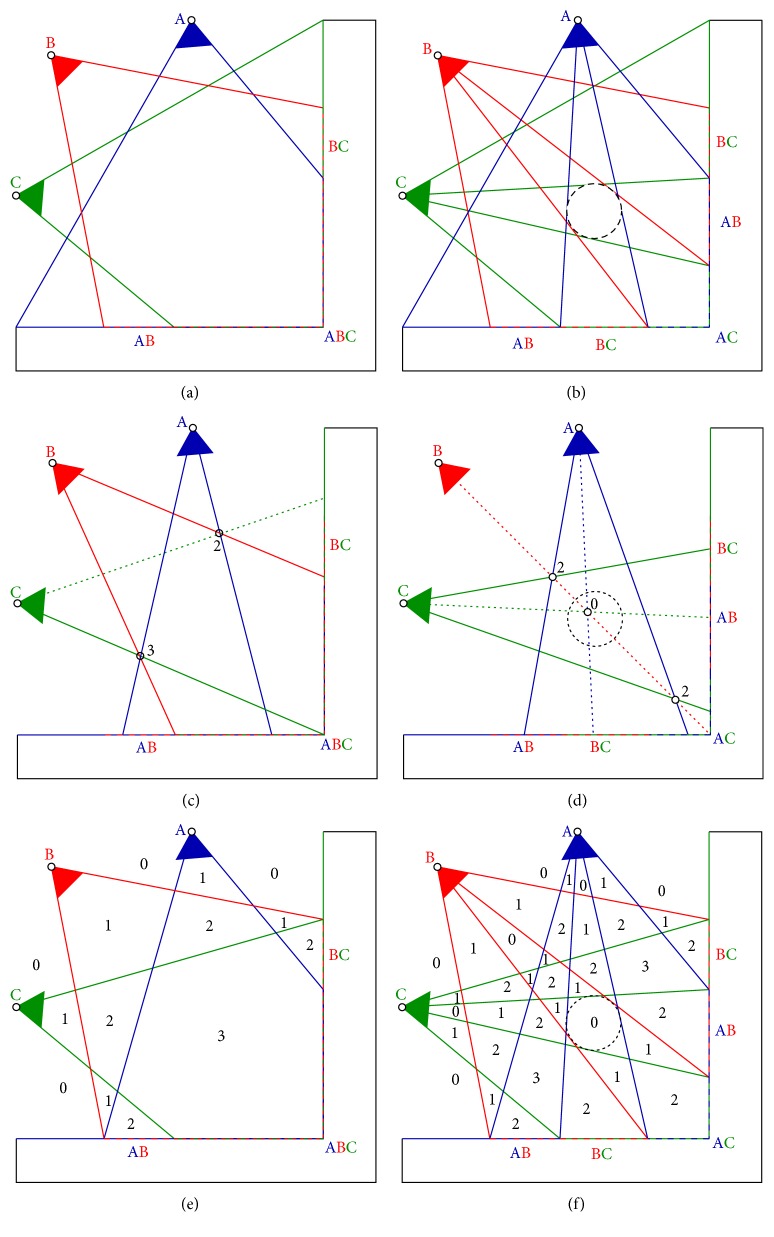
Occluder reveals itself through occlusion. Three sensors with centers A, B, and C, shown as blue, red, and green wedges, observe an L-shaped (black) object. Each ray *r* from a sensor center *O* to a reconstructed point *X* indicates that the space along the line segment *OX* is free. See the text for a more detailed explanation.

Figure 1(a) shows a two-dimensional schematic illustration of three sensors (blue, green, and red wedges) with their respective fields of view. The sensors observe the corner of an L-shaped object. Some points of the object are not observed by any sensor (shown as the solid black line), some are observed by one sensor, some are observed by two sensors (line segments marked by AB and by BC), and some are observed by all three sensors (corner marked by ABC). For simplicity, we assume that all points of the L-shaped object that are seen by at least two sensors are on the input. Note that we are assuming continuous case in this example. Therefore, the number of input points is infinite.

Let us next add a circular object as an occluder to the scene, Figure 1(b), which has no point in the input points. We can still reconstruct the same surface of L-shaped object as in Figure 1(a), but all points are now visible from only (and exactly) two sensors. It is important to notice here that while the set of input points has not changed, the visibility information of the input points has changed. The visibility information has changed in the way that an input point that is occluded by the occluder is not seen by the sensors in which it is occluded.

Let us now introduce a new concept that conveniently captures the effect of having an occluder in the scene. For each point in the space we can construct a measure of its emptiness. We will call this measure* free-space support*. Consider the point marked by “3” in Figure 1(c). The emptiness of this point is supported by three observations. There are three line segments passing through this point that connect the sensor centers A, B, and C with the respective input points. Hence, the free-space support of the point is equal to three. The emptiness of the point marked by “2” is supported only by two observations. The ray from sensor C through the point 2 does not end in an input point. It ends in a point seen by only one sensor: sensor C. Hence the free-space support of this point is equal to two. Note that just points that are seen by at least two sensors are on the input.

After the introduction of the occluder into the scene, Figure 1(d), the free-space support decreases at some points because some of the rays get blocked by the occluder.

Figure 1(e) shows the space partitioned by sensor visibility cones into regions with constant free-space support, which is denoted by black numbers.  Figure 1(f) shows the same after introducing the occluder. We see that a region with zero free-space support surrounded by nonzero free-space support emerged in the scene center. Such a region provides evidence of an occluder even when no point on the occluder has been on the input.

We can consider the space with nonzero free-space support to be free and the complement to be full. Therefore, evidence of the occluder interface can be detected by nonzero to zero change of the free-space support in this ideal noiseless, outlier-less, and continuous case. Additionally, the L-shaped object has zero free-space support and nonzero to zero change is evidence for the L-shaped object interface as well. Therefore, we can say that nonzero to zero change of the free-space support is interface evidence for all objects.

*Hallucinations.* Figure 1(f) shows that there is another nonzero to zero change of the free-space support that is not an evidence for any object. It is the zero to nonzero change of the free-space support at the boundaries of visibility cones. We call these types of evidence* hallucinations*. Hallucinations cause problems in datasets where the scene is not captured from all sides.

### 3.1. Space Discretization by Delaunay Tetrahedralization

It is impossible to compute the free-space support for all points of the space. To be practical, the space is discretized into tetrahedra constructed by the Delaunay tetrahedralization [34] and the free-space support is evaluated for the tetrahedra. The space discretization is constructed by tetrahedralizing the* union of the input points and sensor centers*. By having the sensor centers in the tetrahedralization, the space between the sensors centers and the input points is covered by the tetrahedralization.

The interface evidence can be easily detected by nonzero to zero change of the free-space support in ideal noiseless, outliers-less, and continuous case. In real-world scenarios, however, the number of input points is finite and the amount of noise and outliers can be significant. Nevertheless, we experimentally show that the interface of a scene object, weakly supported as well as strongly supported by input points, can be detected by measuring large free-space support change of nearby tetrahedra. Let us introduce several notations first.

*Tetrahedralization*. We denote a tetrahedron of the Delaunay tetrahedralization of the input points as *T*.

*The Unit σ*. We introduce a constant *σ* that equals 2.0 times median of all edges lengths of all tetrahedra. The value *σ* represents the smallest reconstructible object.

*Input Points and Segments (Lines) of Sight*. We assume that we have on the input a set *C* of sensor centers and a set *S* of pairs (*c*, *p*) ∈ *S* where *c* ∈ *C* and *p* is an input 3D point. The set *S* is a set of input points augmented with visibility information. We call the line segment defined by 3D points (*c*, *p*) ∈ *S* the segment of sight or simply segment. We call the line defined by 3D points (*c*, *p*) ∈ *S* the line of sight. We call input points augmented with visibility information, that is, the set *S*, the input segments of sight.

The set of input points is denoted as *P*(*S*) = {*p*∣(*c*, *p*) ∈ *S*}. Note that one input point *p* can be augmented (and typically is) with multiple sensor centers *c*.

*Input Point Weights*. We assign to each of the input points *p* a weight denoted by *α*(*p*). The weight *α*(*p*) ≥ 1 reflects the amount of input points in *σ* surrounding the point *p*.

*Free-Space Support*. Free-space support *f* is a measurement of emptiness. Roughly speaking, if a sensor with center *c* sees a point *p*, then the segment of sight (*c*, *p*) ∈ *S* supports the emptiness of space between *c* and *p*. The free-space support *f*(*T*) of tetrahedron *T* is computed from segments (*c*, *p*) ∈ *S* intersecting *T* as

(1) f(T)=∑(c,p)∈STα(p)with  ST={(c,p)∈S ∣ (c,p)∩T≠∅},

where (*c*, *p*)∩*T* denotes the intersection of the line of sight (*c*, *p*) and the tetrahedron *T*.

**Figure 2:**
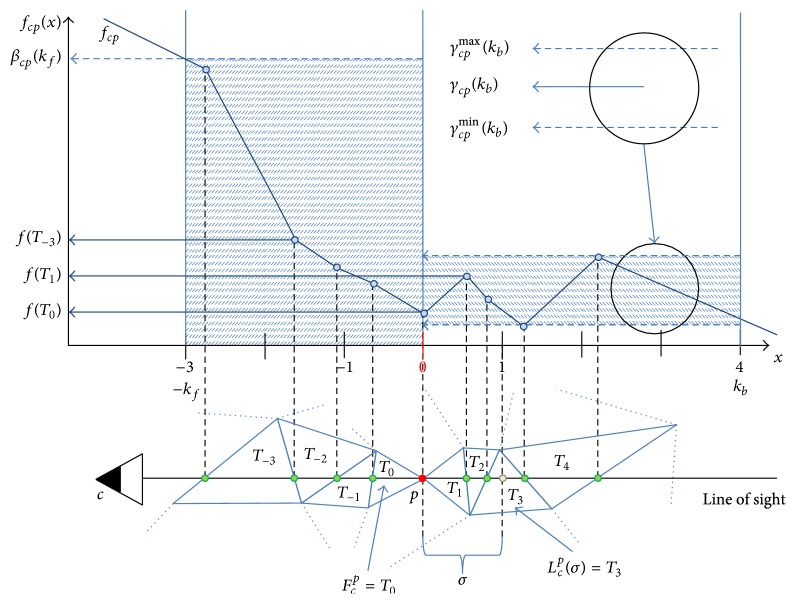
Free-space support on line of sight. Illustration of functions *f*_*cp*_(*x*), *β*_*cp*_(*k*), and *γ*_*cp*_(*k*). See text for more details.

*Free-Space Support on a Line of Sight*. We denote free-space support on line of sight (*c*, *p*) ∈ *S* as *f*_*cp*_.  Figure 2 illustrates how the free-space support on a line of sight is computed. The function *f*_*cp*_(*x*) is a piecewise linear function that interpolates the free-space supports *f*(*T*_*j*_) of tetrahedra *T*_*j*_ crossed by the line of sight (*c*, *p*) ∈ *S* (bottom). Note that the domain of the function *f*_*cp*_ is in units *σ*. We introduce the following notations to measure extremal values of the function *f*_*cp*_ on specific intervals:

(2) βcp(k)=max⁡⁡(fcp(x) ∣ x∈〈−k,0〉),γcp(k)=(max⁡⁡(fcp(x) ∣ x∈〈0,k〉)2iiiiiii+min⁡⁡(fcp(x) ∣ x∈〈0,k〉))2.

The function *β*_*cp*_(*k*) measures the maximal value of *f*_*cp*_(*x*) in range 〈−*k*, 0〉, that is, in *kσ* distance from the point *p* towards the sensor *c*. The function *γ*_*cp*_(*k*) measures the average of maximal and minimal values of *f*_*cp*_(*x*) in range 〈0, *k*〉, that is, in *kσ* distance from the point *p* away from the sensor *c*. See Figure 2.

*Free-Space Support Jump*. We define the following functions to be able to measure free-space support change in close surrounding of an input point:

(3) $$\begin{array}{c} \epsilon_{cp}^{\text{abs}}\left(k_{f},k_{b}\right)=\beta_{cp}\left(k_{f}\right)-\gamma_{cp}\left(k_{b}\right),\\ \epsilon_{cp}^{\mathrm{rel}}\left(k_{f},k_{b}\right)=\frac{\gamma_{cp}\left(k_{b}\right)}{\beta_{cp}\left(k_{f}\right)}. \end{array}$$

The function *ϵ*_*cp*_^abs^ evaluates the absolute change of the free-space support on the line of sight (*c*, *p*) in the 〈−*k*_*f*_ *σ*, *k*_*b*_ *σ*〉 surrounding the input point *p*. The function *ϵ*_*cp*_^*rel*⁡^ evaluates a relative change.

The functions are chosen this way because we want to be able to detect big change in free-space evidence near some 3D point. However, measuring just relative change is not enough because relative change of small (noise) numbers can be also significant. Therefore we study combination of absolute and relative change.

*Outliers Level*. If a point *p* represents a real interface point, then the value of the function *γ*_*cp*_ represents the amount of outlier lines of sight (*c*, *p*) ∈ *S* crossing the corresponding part of full space. The function reflects average free-space evidence accumulated in the space that should be occupied by an object. Only an outlier that is seen by a sensor but should be occluded by the object can contribute to this evidence; therefore the function *γ*_*cp*_ reflects the outliers level.

*The First and the Last Crossing Tetrahedron*. The first crossing tetrahedron of a line segment (*c*, *p*) is denoted by *F*_*c*_^*p*^. It is the tetrahedron containing point *p*, the tetrahedron *T*_0_ on Figure 2. We denote as *L*_*c*_^*p*^(*d*) the tetrahedron (*L*_*c*_^*p*^(*σ*) = *T*_3_ on Figure 2) that contains the point $p+d\overset{⃗}{\left(c,p\right)}\in L_{c}^{p}(d)$ (gray point for *d* = *σ* on Figure 2).

## 4. Experimental Evaluation of the Free-Space Support Properties

In this section we experimentally evaluate how the free-space support of tetrahedra looks in free and full space near ground-truth weakly and strongly supported surfaces in a real-world scenario. We evaluate it under controlled levels of noise and controlled levels of undersampling. More specifically, we evaluate *f*_*cp*_(*x*) at 25 discrete points *x* ∈ {−12,12}. Note that the domain of the function *f*_*cp*_ is in *σ* units.

**Figure 3:**
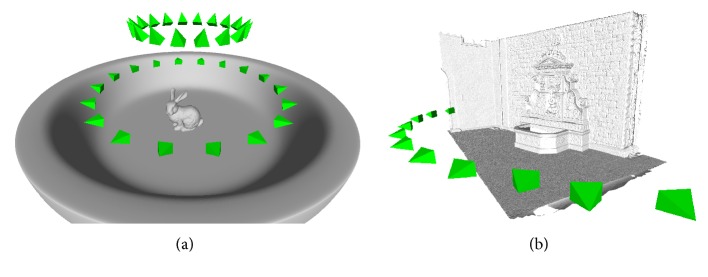
Ground-truth datasets: (a) rendering of the mesh and sensors positions of the “bunny” dataset. 36 sensors in two elevations are placed in the scene. Ground truth for each sensor is computed directly from the mesh. Random undersampling and noisifying of the bunny object (without plate) and outlier injection are used at different levels to generate depth maps for quantitative analysis. (b) Ground-truth laser scan of the “fountain” dataset and the related 11 cameras (registered to the scan).

We evaluate the free-space support in two scenes (see Figure 3). The first scene is a synthetically created scene of a bunny on a plate. We have downloaded the publicly available bunny dataset from the Stanford 3D Scanning Repository (see http://graphics.stanford.edu/data/3Dscanrep/) and we have created the plate in a 3D modeling software. We have uniformly distributed 36 sensors on two circles in two elevations around the bunny so that each sensor sees the whole bunny and, more importantly, each sensor is oriented so that the plate is visible all around the bunny. In another words, the whole bunny occludes a part of the plate in each view.

The plate is always strongly supported. The bunny object is represented by 160 K of input points and the plate object is represented by 2.4 M of input points. We have created three datasets from the bunny on the plate scene with the 36 sensors:

(i) original scene without undersampling and without outliers,
(ii) bunny undersampled to 3% of the original points plus 30 K outliers,
(iii) bunny undersampled to 3% of the original points plus 130 K outliers.

The second scene is a real-world scene of a “fountain.” The “fountain” dataset is a benchmark dataset [6] provided by Strecha et al. and contains 11 calibrated images with a resolution of 3072 × 2048 together with ground-truth laser scan. We have computed input segments of sight by MVS plane-sweeping technique described in [33]. We consider input points that are within 2*σ* distance to the ground-truth laser scan to be ground-truth input points.

We denote the input segments of sight, that is, the set *S* of the “bunny” (i–iii) and “fountain” datasets as BunnySSS, BunnyWSS, BunnyWSSO, and FountainSSS, where SSS stands for strongly supported surface, WSS stands for weakly supported surface, and WSSO stands for weakly supported surface with large amount of outliers.

Note that in all the datasets we know exactly which points are ground-truth interface points and in the “Bunny” datasets we additionally know which ground-truth points are from the bunny object surface and from the plate object surface. We also know which points are located inside or outside the object, that is, in full or free space.

In this section we use the following notation: *SUR*(*S*)⊆*S* is the set of input segments of sight that contain ground-truth interface points of the bunny without the plate (of the fountain), *FREE*(*S*)⊆*S* is the set of input segments of sight that contain input points that are located in the free space, and *FULL*(*S*)⊆*S* is the set of input segments of sight that contain input points that are located in the full space.

### 4.1. Free-Space Support Evaluation

**Figure 4:**
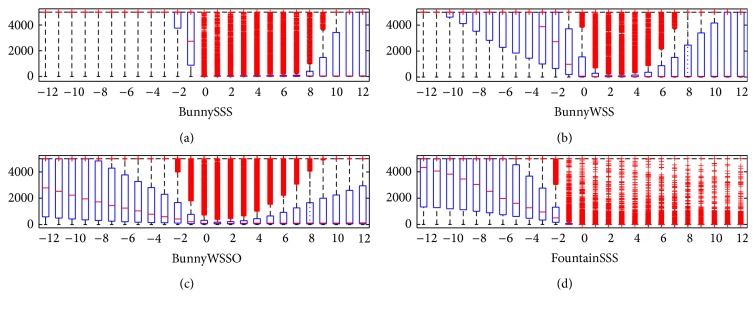
Free-space support evaluation. Free-space support at different distances near the ground-truth surface points *p* ∈ *P*(*SUR*(*S*)) for *S* ∈ {BunnySSS, BunnyWSS, BunnyWSSO, and FountainSSS} datasets. We compute a set of free-space supports {*f*_*cp*_(*x*)∣(*c*, *p*) ∈ *SUR*(*S*)} for each point *x* ∈ {−12,…, 12} and visualize it using the Matlab boxplot function (see text for more details).

In this section we evaluate *f*_*cp*_(*x*) at points {−12,…, 12} just for the ground-truth interface points *p* ∈ *P*(*SUR*(*S*)).  Figure 4 shows the Matlab boxplots of values {*f*_*cp*_(*x*)∣(*c*, *p*) ∈ *SUR*(*S*)} for each *x* ∈ {−12,…, 12}. It can be seen that the free-space support between the observer and the surface, for example, *x* ∈ {−3,…, 0}, is in the majority of cases relatively large compared to the free-space support for *x* ∈ {1,…, 4}; that is, free-space support is large in free space. Moreover, it can be seen that the free-space support is relatively small at a small distance behind the surface, *x* ∈ {1,…, 4}; that is, the free-space support is small in full space. This property holds for all datasets. It holds for weakly supported as well as for strongly supported surfaces. Therefore, it is reasonable to measure the free-space support jump in 〈−3*σ*, 4*σ*〉 surrounding the input points.

Results in Figure 4 are represented by the Matlab boxplot (see http://en.wikipedia.org/wiki/Box_plot) function which shows values 25% to 75% quantile as a box with a horizontal line at the median. The red crosses show data beyond 1.5 times the interquartile range.

### 4.2. Free-Space Support Jump Evaluation

Based on the experiments in the previous section, we choose *k*_*f*_ = 3 and *k*_*b*_ = 4. We evaluate absolute *ϵ*_*cp*_^abs^(*k*_*f*_, *k*_*b*_) and relative *ϵ*_*cp*_^*rel*⁡^(*k*_*f*_, *k*_*b*_) free-space support jump on ground-truth interface input points *p* ∈ *P*(*SUR*(*S*)), input points located in ground-truth free space *p* ∈ *P*(*FREE*(*S*)), and input points located in ground-truth full space *p* ∈ *P*(*FULL*(*S*)). We introduce the two interface classifiers *K*_*rel*⁡_(*c*, *p*) and *K*_abs_(*c*, *p*) in order to capture properties of the free-space support jump functions. Given an input line of sight (*c*, *p*) ∈ *S* the classifiers classify the point *p* as being interface point INT or noninterface point NOI according to the following rule:

(4) $$\begin{array}{c} K_{\mathrm{rel}}\left(c,p\right)=\{\begin{array}{cc} \text{INT} & \text{for}\;\;\left(\epsilon_{cp}^{\mathrm{rel}}\left(k_{f},k_{b}\right)<k_{\mathrm{rel}}\right)\\ \text{NOI} & \text{otherwise} \end{array}\\ K_{\text{abs}}\left(c,p\right)=\{\begin{array}{cc} \text{INT} & \text{for}\;\left(\epsilon_{cp}^{\text{abs}}\left(k_{f},k_{b}\right)>k_{\text{abs}}\right)\;\\ \text{NOI} & \text{otherwise,} \end{array} \end{array}$$

where *k*_*f*_, *k*_*b*_, *k*_*rel*⁡_, and *k*_abs_ are parameters of the classifiers *K*_*rel*⁡_(*c*, *p*) and *K*_abs_(*c*, *p*).

**Figure 5:**
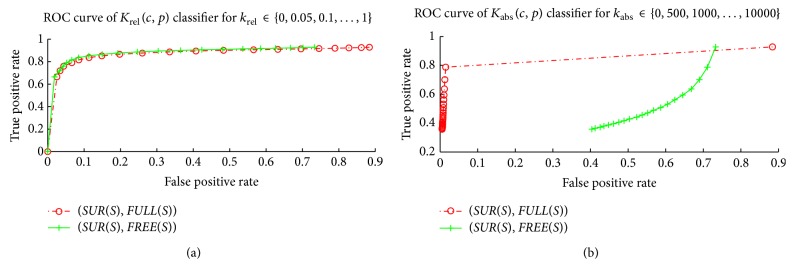
Free-space support jump evaluation. (a) relatively large number of true positives at the price of up to 10% of false positives can be detected using the relative free-space support jump classifier *K*_*rel*⁡_(*c*, *p*). (b) absolute free-space support jump classifier *K*_abs_(*c*, *p*) can be used for detecting input points that are located in full space. The set *SUR*(*S*) is considered positive examples and the set *FREE*(*S*) (*FULL*(*S*)) negative examples. See text for more details.

Figure 5 shows receiver operating characteristic curves

(ROC see (http://en.wikipedia.org/wiki/Receiver_operating_characteristic)) of the classifiers *K*_*rel*⁡_(*c*, *p*) and *K*_abs_(*c*, *p*). We evaluate classifier *K*_*rel*⁡_(*c*, *p*) (*K*_abs_(*c*, *p*)) on a set *A* of segments of sight (*c*, *p*) ∈ *A* for classifier parameters *k*_*f*_ = 3, *k*_*b*_ = 4, and *k*_*rel*⁡_ ∈ {0,0.05,0.1,…, 1.0} (*k*_abs_ ∈ {0,500,1000,…, 10000}).

For given *k*_*rel*⁡_ (*k*_abs_) we compute the rate of the results classified positively as INT out of the positive examples *A* = *SUR*(*S*), that is, the true positive rate. For the same *k*_*rel*⁡_ (*k*_abs_) we compute the rate of the results classified positively as INT out of the negative examples *A* = *FULL*(*S*) (*A* = *FREE*(*S*)), that is, the false positive rate. Finally, we plot the (false positive rate, true positive rate) as point of the ROC. We have used the set *S* of all segments of sights from all datasets; that is, *S* = BunnySSS ∪ BunnyWSS ∪ BunnyWSSO ∪ FountainSSS.

Figure 5(a) shows that the classifier *K*_*rel*⁡_(*c*, *p*) can detect relatively large number, that is, 84% of interface input points, correctly for the price of up to 10% of wrongly classified noninterface input points. We have evaluated that, if we allow 10% of false positives, then the parameter that gives the maximal percentage of true positives is *k*_*rel*⁡_ = 0.3. The classifier *K*_*rel*⁡_(*c*, *p*) with this parameter *k*_*rel*⁡_ = 0.3 gives 84% of true positives. The smaller the parameter *k*_*rel*⁡_ is, the lower the percentage of the false positives the classifier *K*_*rel*⁡_(*c*, *p*) gives at the price of lower true positives; that is, a conservative approach to the *k*_*rel*⁡_ parameter setting is to set a smaller value of the *k*_*rel*⁡_ parameter.

Figure 5(b) shows that the classifier *K*_abs_(*c*, *p*) can be used to detect the input point that is located in the full part of the space. We have evaluated that, if we allow just 2% of false positives for *A* = *FULL*(*S*), then the parameter that yields the maximal percentage of true positives is *k*_abs_ = 500. The classifier *K*_abs_(*c*, *p*) with this parameter *k*_abs_ = 500 gives 79% of true positives. The larger the parameter *k*_abs_ is, the lower the percentage of false positives the classifier *K*_abs_(*c*, *p*) yields at the price of lower true positives; that is, a conservative approach to the *k*_abs_ parameter setting is to set a larger value of the *k*_abs_ parameter.

### 4.3. Outlier Level Evaluation

We evaluate the level of outliers *γ*_*cp*_^max⁡^(*k*_*b*_) in the same way we have evaluated free-space support jump in the previous section. We use the following classifier:

(5) $$\begin{array}{c} K_{\text{outl}}\left(c,p\right)=\{\begin{array}{cc} \text{INT} & \text{for}\;\;\left(\gamma_{cp}\left(k_{b}\right)<k_{\text{outl}}\right)\\ \text{NOI} & \text{otherwise,} \end{array} \end{array}$$

where *k*_*b*_ and *k*_outl_ are parameters of the classifier *K*_outl_(*c*, *p*). We evaluate the classifier *K*_outl_(*c*, *p*) for *k*_outl_ ∈ {0,50,100,…, 1000}.

**Figure 6:**
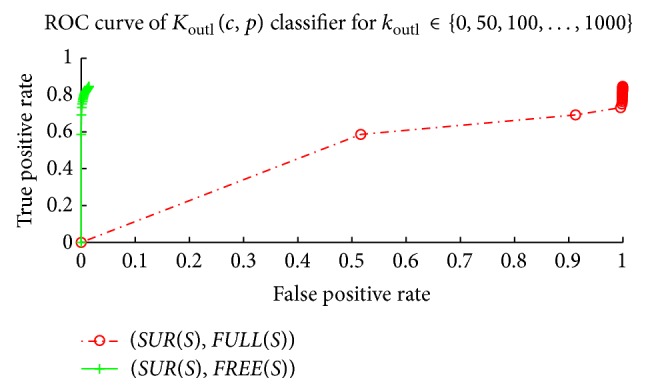
Outliers level evaluation. Outliers level classifier *K*_outl_(*c*, *p*) can be used for detecting input points that are located in the free space. The set *SUR*(*S*) is considered positive examples and the set *FREE*(*S*) (*FULL*(*S*)) negative examples. See text for more details.

Figure 6 shows that the classifier *K*_outl_(*c*, *p*) can be used to detect an input point that is located in the free space. We have evaluated that, if we allow just 1% of false positives for *A* = *FREE*(*S*), then the parameter that yields the maximal percentage of true positives is *k*_outl_ = 700. The classifier *K*_outl_(*c*, *p*) with this parameter *k*_outl_ = 700 yields 83% of true positives. The smaller the parameter *k*_outl_ is, the lower the percentage of false positives the classifier *K*_outl_(*c*, *p*) gives at the price of lower true positives; that is, a conservative approach to the *k*_outl_ parameter setting is to set a smaller value of the *k*_abs_ parameter.

## 5. Interface Classifier

In this section we design a new interface classifier. The classifier takes into account the free-space support jump and outliers level. Based on the experiments in the previous sections we can say that a large free-space support jump, coupled with a low outliers level in 〈−3*σ*, 4*σ*〉 surrounding an input point, is good evidence for an interface. Given an input line of sight (*c*, *p*) ∈ *S* the following classifier *K*(*c*, *p*) classifies the point *p* as being an interface point INT or a noninterface point NOI:

(6) K(c,p)={INTfor  (ϵcprel⁡(kf,kb)<krel⁡)iiiiii∧(ϵcpabs(kf,kb)>kabs)iiiiii∧(γcp(kb)<koutl)NOIotherwise,

where *k*_*f*_, *k*_*b*_, *k*_*rel*⁡_, *k*_abs_, and *k*_outl_ are parameters of the classifier *K*(*c*, *p*) and are constant in all of our experiments. All parameters *k*_*f*_, *k*_*b*_, *k*_*rel*⁡_, *k*_abs_, and *k*_outl_ were discussed and evaluated experimentally in the previous section.  Table 1 summarizes computed and used values. The used values were chosen using a conservative approach, that is, lower percentage of false positives at the price of lower true positives.

Table 2 shows the decision rates of the interface classifier on sets *S* ∈ {BunnySSS, BunnyWSS, BunnyWSSO, and FountainSSS} of input segments of sight. We consider the set *SUR*(*S*) to be positive examples (i.e., “interface”) and the set *FREE*(*S*) ∪ *FULL*(*S*) to be negative examples (i.e., “noninterface”).

Based on the experiments, we can say that, if the classifier classifies a point as INT, then it is most likely an interface point in the real world. Note that this property holds for weakly supported surfaces as well. On the other hand if the classifier classifies a point as NOI, we cannot say that it is not an interface point in the real world. In the next sections we show how we use the interface classifier to modify the state-of-the-art method [24], which is not capable of reconstructing weakly supported surfaces, so that it will obtain the ability to reconstruct weakly supported surfaces. Although the number of true positives is around 50%, we will show that it is enough for the new method to reconstruct weakly supported surfaces.

## 6. Finding Surfaces by Solving a Graph Optimization Problem

We construct* s-t graph *(*STG*) (*V*, *E*) for given Delaunay tetrahedralization of the input points in the same way as described in [24, 33]. We denote tetrahedron *T* that corresponds to a node *v* ∈ *V* as *T*(*v*). We denote oriented face *F* of the tetrahedralization that corresponds to an oriented* v*-edge *e* ∈ *E* as *F*(*e*). Given the graph DG and weights *w*(*e*) of all of its edges *e* ∈ *E* an *s*-*t*-cut  *C*(*𝕊*, *𝕋*) is a partition of *V* into two disjoint sets *𝕊* and *𝕋* where *s* ∈ *𝕊* and *t* ∈ *𝕋*. The *C*(*𝕊*, *𝕋*) is defined as follows:

(7) C(S,T)=∑e=(u,v) ∣ u,v∈S∖{s}w(e)+∑e=(s,v) ∣ v∈S∖{s}w(e) +∑e=(v,t) ∣ v∈T∖{t}w(e).

The minimum* s*-*t*-cut optimization problem is the minimization of the *s*-*t*-cut value *C*(*𝕊*, *𝕋*). There are many classical algorithms that efficiently solve this problem [36]. Following [24], we cast the surface reconstruction problem as the minimal *s*-*t*-cut problem of the graph STG. We interpret the final sets *𝕊* and *𝕋* as that tetrahedra *T*(*v*)∣*v* ∈ *𝕊* are labeled as being free and tetrahedra *T*(*v*)∣*v* ∈ *𝕋* are labeled as being full. The final surface is then reconstructed as the union of oriented faces (triangles) of the STG that are on the full-free interface. The surface is therefore guaranteed to bind a volume, that is, to be watertight and self-nonintersecting.

## 7. Using an Interface Classifier to Reconstruct Weakly Supported Surfaces

We have introduced a way how to detect occluder just from the accumulated free-space evidence in previous sections. Now we use this tool to reconstruct them. Our idea is to modify weights of the STG such that the positively classified occluder tetrahedra will be labeled as full after the minimum* s*-*t*-cut optimization. The natural way how to do it is to set weight of corresponding* t*-edges to infinity. However, we do just enforce them instead. By enforcing the* t*-weights we mean that we enlarge the values of the weights of the* t*-edges.

This will force the optimization to label the occluder nodes as full and the weakly supported surface will be reconstructed accordingly. It is important to note that we do not have to enforce all occluder tetrahedra. It is enough to enforce just few. The reason is nicely explained in the paper [33]. Therefore, besides the fact that the number of true positives of our classifier is not 100%, it is far enough for weakly supported surfaces reconstruction.

Following [33], all we need to do in order to preserve the weakly supported surface is to enforce the* t*-weights of nodes *v* ∈ *V* where tetrahedra *T*(*v*) are located inside the occluder.

We proceed sequentially. In the first iteration we compute the weights of STG in the same way as in [24] but we use adaptive *α*(*p*) weight of input point *p* (proposed in [33]). We compute the free-space support as was described in previous sections. Then we evaluate the interface classifier and, for the segments of sight classified as INT (interface), we enforce* t*-weight in the place we assume is inside of the object. Weight *w*(*e*) of a* t*-edge *e* = (*v*, *t*) ∈ *E* is enforced to

(8) w(e)=w(e)∑(c,p)∈SK(v)ϵcpabs(kf,kb)with  SK(v)={(c,p)∈S ∣ p∈Lcp(kbσ)iiiiiiiiiiiiiiiiiiiii∧Lcp(kb)=T(v)∧K(c,p)=INT{(c,p)∈S ∣ p∈Lcp(kbσ)∧Lcp(kb)}.

Algorithmic overview and the implementation details of the proposed method are in Section 8. Finally, in Section 9 we provide an experimental evaluation of the accuracy and the ability of reconstruction of weakly supported surfaces on synthetic as well as on real-world datasets and compare them to other methods.

### 7.1. Comparison to Related Work

Both the newly proposed method and the method proposed in [33] differ from the method proposed in [24] in how the STG edges weights are computed. First, the newly proposed method and the method proposed in [33] use adaptive *α*(*p*) weight of input point *p* while the method proposed in [24] uses a constant weight for each input point *p*. Second, the newly proposed method and the method proposed in [33] use an interface classifier to enforce* t*-weights in order to be able to reconstruct weakly supported surfaces. The method proposed in [33] does not define the interface classifier explicitly. However, [33] uses a decision rule that can be formulated as interface classifier *K*_jan_ as follows:

(9) Kjan(c,p)  ={INTfor  (φ(Lcp(σ))φ(Fcp)<0.5)∧(φ(Lcp)<1000)NOIotherwise,

where

(10) $$\begin{array}{c} \varphi \left(T\left(u\right)\right)=\varphi \left(u\right)=\underset{e=\left(u,v\right)\in E_{v}}{\sum }w\left(e\right). \end{array}$$

If *σ* equals zero, then *f*(*T*(*u*)) = *φ*(*T*(*u*)); that is, if *σ* = 0, then the free-space support of the tetrahedron *T*(*u*) equals the sum of the weights of all the incoming edges to the node *u*.

We define *φ*_*cp*_(*x*) on the line of sight (*c*, *p*) ∈ *S* for the function *φ* in the same way as the free-space support weight *f*_*cp*_(*x*) on the line of sight (*c*, *p*) ∈ *S* for the function *f* is defined.

The major advantage of the newly proposed method with respect to the method proposed in [33] is that it produces much more accurate results on strongly supported surfaces and reconstructs weakly supported surfaces better as shown in experiments in Section 9.

**Figure 7:**
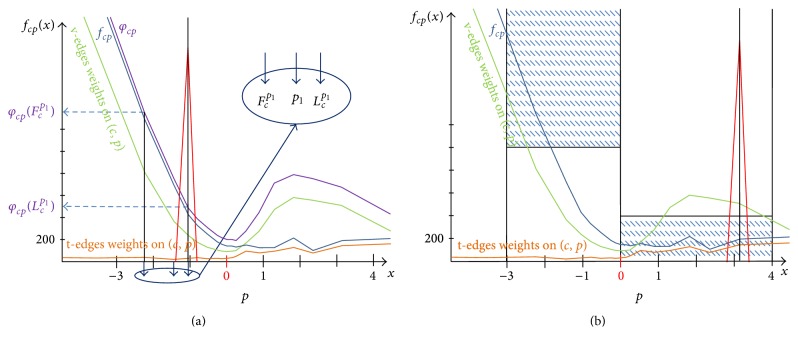
New interface classifier *K* versus *K*_jan_ proposed in [33]. (a, b) Point *p* (*x* = 0) represents a real interface point of a ground-truth weakly supported surface. (a) illustrates the typical situation when classifier *K*_jan_ wrongly classifies a point *p*_1_ as an interface point and enforces* t*-weight (red peak) in wrong place, that is, in space that should be labeled as free. (b) Design of the new interface classifier *K* leads to correct classification of interface point *p* and* t*-weight enforcement in correct place. Note that the* t*-edge weights are low because this is illustration of weights near a weakly supported surface.

*Why Is the Newly Proposed Method More Accurate Than the Method Proposed in [33]?* Figure 7(a) shows a typical situation where the classifier *K*_jan_ wrongly classifies the point *p*_1_ as an interface point. While the free-space support decreases rapidly in front of the point *p*_1_, which is in front of a real surface point *p* (*x* = 0), the classifier *K*_jan_ classifies the point *p*_1_ as interface point and enforces the* t*-weight behind *p*_1_ (red peak), which leads to less accurate result. However, proposed interface classifier *K* searches for such point *p* and an associated sensor *c* for which the free-space support function *f*_*cp*_ is rapidly decreasing at some (reasonably) large distance *k*_*f*_ in front of the point *p* (i.e., towards the sensor *c*). Additionally it is reasonably small and almost constant at (reasonably) large distance *k*_*b*_ behind the point *p* (i.e., backwards the sensor *c*); see Figure 7(b). Afterwards, based on the positive interface classification we enforce* t*-weight in distance *k*_*b*_ (deep enough) behind the point *p* in order to ensure that it is enforced inside of the real object (occluder).

## 8. Implementation Details

We use the Computational Geometry Algorithms Library (CGAL) (see http://www.cgal.org/) [37] to create the tetrahedralization. The tetrahedralization is created incrementally. When adding a new point *o* from the input point cloud we first check if there is a vertex *p* of the actual tetrahedralization within a certain distance from *o*. The distance is set in terms of the *o* and *p* reprojection distance in pixels to a value in the range 〈2,10〉 in order to be able to fit the data into memory. If there is such a vertex *p*, then we do not add *o* to the tetrahedralization but associate the sensor with the vertex *p* and increase *α*(*p*) by 1. Otherwise, we will add the point *o* to the tetrahedralization, associate the sensor with it, and initialize *α*(*o*) to 1. The approach is similar to [38]. Each vertex has a set of sensors and an *α*(*p*) value associated with it. The vertices of the tetrahedralization are considered input points, associated sensors are considered to be visibility information of input points, and *α*(*p*) is the weight of the input point *p*.

Implementation of the proposed method is described in Algorithm 1. We build the* s*-*t* graph. Next, we compute the weights of all edges. Our approach to setting up the weights of the graph is sequential. In the first part, we compute all weights of all edges as described in Section 7 (line 2 in Algorithm 1). In the second part, we evaluate on each input point *p* and each associated sensor *c* the newly proposed classifier *K*(*c*, *p*). If the classification result is INT, then we multiply the weight of the* t*-edge (*v*, *t*) where *L*_*c*_^*p*^(*k*_*b*_ *σ*) = *T*(*v*) by *ϵ*_*cp*_^abs^(*k*_*f*_, *k*_*b*_) value (lines 3–8 in Algorithm 1).  Table 1 shows classifier parameters, which we use in all of our experiments. Finally, we solve the minimal* s*-*t* cut problem using the software (see http://pub.ist.ac.at/~vnk/software.html) described in [36] (line 9 in Algorithm 1). Finally, we do two steps of a Laplacian based smoothing (line 11 in Algorithm 1) as in [24]. We focus just on creating an initial surface, which can be later refined as in [35].

Table 3 shows the running times of different parts of Algorithm 1 for different datasets. Table 3 shows that the second part *t*_*o*_2__ is 2 to 10 times faster than the first one *t*_*o*_1__. Iterations in all parts are performed parallel in our implementation. Therefore, all parts are relatively fast.

## 9. Experimental Results

All experiments were computed on Intel Core i7 CPU machine with NVIDIA 285GTX graphics card, 12 GB RAM, and 64-bit Windows 7 OS. We use several different calibrated datasets in this paper: “bunny,” “Lausanne,” “castle,” “herzjesu,” “fountain,” and “googleStreetView.” The “bunny” and “fountain” datasets are described in Section 4. The street view dataset “Lausanne” was provided by Christopher Strecha and contains 1514  2000 × 1500 images.

The street view data set “goolgeStreetView” is Google street view Pittsburgh experimental image set [39]. The first 10000 camera matrices were obtained by the method proposed in [40]. We have to point out that the quality of images (compared to the quality of images from a DLSR or compact camera) was poor in this dataset. The calibration was not optimal either because the calibration was done on spherical images where the internal parameters of each perspective camera out of 6 Ladybug perspective cameras are unknown or because in the proposed pipeline we use 6 perspective cutouts from one spherical image instead of original perspective images from the Ladybug device. Therefore, we consider the “googleStreetView” dataset the challenging one.

We compute the depth map for each of the 1500 (10000) images from “Lausanne” (“googleStreetView”) dataset but to reconstruct different parts of the city we choose 3D points from different volumes parts defined by boxes in order to fit into memory.

The “castle,” “herzjesu,” and “fountain” datasets are the benchmark datasets [6]. The “castle” dataset contains 30  3072 × 2048 images, the “herzjesu” dataset contains 8 3072 × 2048 images, and the “fountain” data set contains 11  3072 × 2048 images.

Let us denote the newly proposed method in this paper as “proposed,” method [33] as “former,” method [24] as “baseline,” and method [23] as “Poisson.”

### 9.1. Approach to Comparison with Ground Truth

Let us describe how we compare a reconstruction to a ground truth. We generate two depth maps *d*_*r*_ for computed 3D mesh and *d*_*g*_ for the ground-truth mesh for each sensor. The depth value for a pixel *x* of a depth map *d* is denoted as *d*(*x*). We evaluate accuracy value acc(*x*, *c*) = |*d*_*r*_(*x*) − *d*_*g*_(*x*)|/*σ* for each sensor *c* and for each pixel *x*.

We compute a histogram from all acc(*x*, *c*) values as follows: bins 1 to 10 represent the histogram of accuracy values acc(*x*, *c*) of all pixels *x* and all sensors *c* where *d*_*r*_(*x*) and *d*_*g*_(*x*) are defined; that is, *d*_*r*_(*x*)>−1∧*d*_*g*_(*x*)>−1. Bin 11 is the number of pixels where acc(*x*, *c*) > 10 and bin 12 is the number of pixels where the ground-truth depth map *d*_*g*_ is defined but the reconstructed depth map *d*_*r*_ is not defined; that is, |{*x*∣*d*_*r*_(*x*) = −1∧*d*_*g*_(*x*) > −1}|.

Therefore, the more accurate result has a higher number in the first bins. The first two bins are authoritative because they contain the majority of the points. We call the aforementioned histogram occupancy histogram. We call the value of a bin *X* occupancy at *σX* or occupancy at bin *X*.

### 9.2. Quantitative Analysis

In this section we provide a quantitative analysis of results of proposed, former, baseline, and Poisson method with respect to the ground truth.

**Figure 8:**
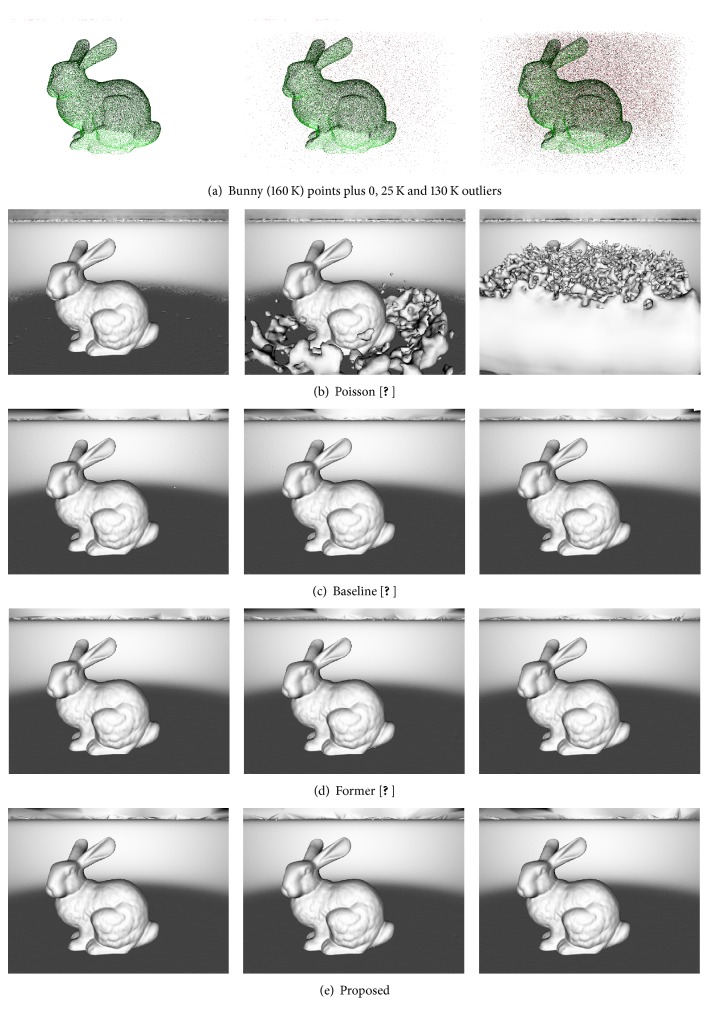
Robustness to outliers. (a) The input points are visualized without plate input points in order to be able to visualize the level of outliers with respect to input points of the bunny. (b), (c), (d), and (e) Results of Poisson, baseline, former, and proposed methods. Baseline, former, and proposed methods are all robust to large number of outliers (130 K) when the surface of the bunny is strongly supported (bunny is sampled by 160 K input points).

*Robustness to Outliers*.  Figure 8 shows robustness of evaluated methods to outliers. It shows that proposed, former, and baseline methods are robust in the presence of 25 K and 130 K randomly distributed outliers in manually selected cube around the bunny. The Poisson method fails completely when 130 K of outliers were added to the scene.

**Figure 9:**
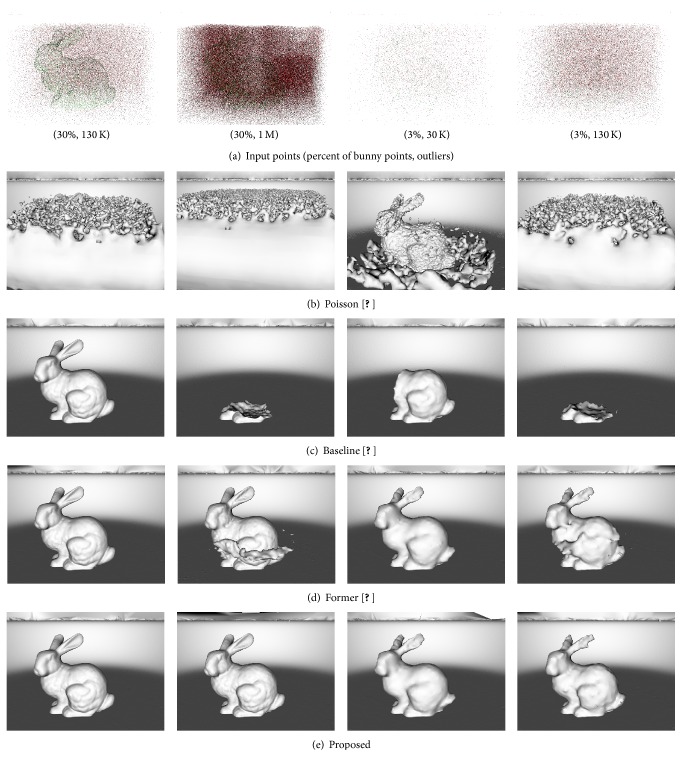
Robustness to undersampling and outliers. (a) The input points are visualized without plate input points in order to be able to visualize the level of outliers with respect to input points of the undersampled bunny. (b), (c), (d), and (e) Results of Poisson, baseline, former, and proposed methods.

*Robustness to Undersampling and Outliers*.  Figure 9 shows the robustness of evaluated methods to undersampling and outliers. We have randomly undersampled just the bunny object (the plate object was not undersampled) to 30% and 3%. We have randomly distributed 130 K and 1 M outliers in manually selected cube around the bunny.  Figure 8 shows that Poisson method has completely failed in all cases. The baseline method was successful just in the first case where the surface is relatively strongly supported by the input points and the outliers level is lower than the surface sampling by the input points. The former method can reconstruct the weakly supported object better than the baseline method; however, it is not perfect in all cases. On the other hand, the proposed method is stable in all cases and it is able to reconstruct weakly supported surfaces perfectly. This experiment also shows that while the TP classification rate showed in Table 2 of the proposed interface classifier (Section 5) is around 50% it is still enough to reconstruct weakly supported surfaces.

**Figure 10:**
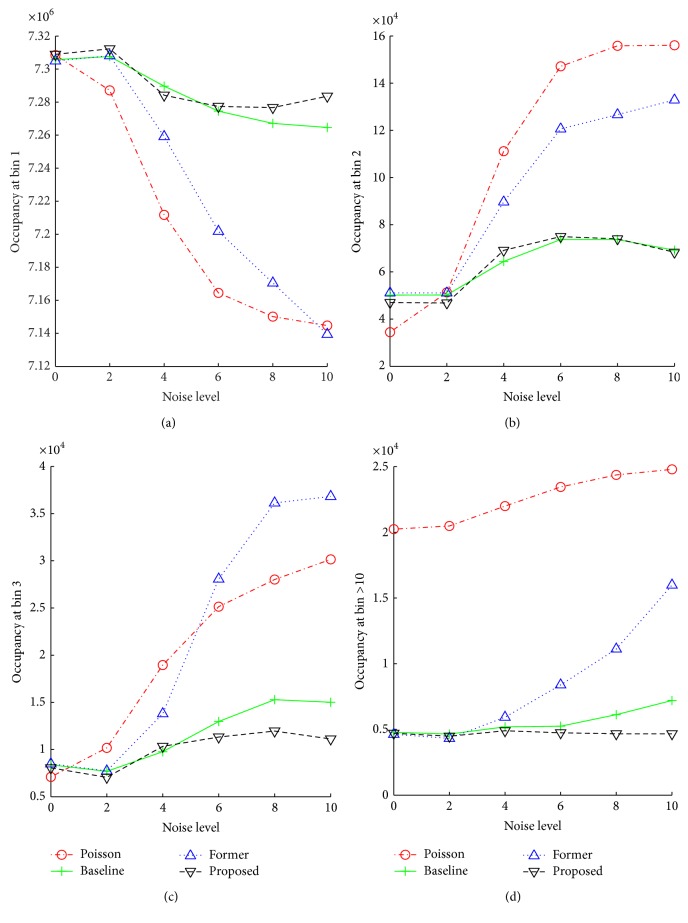
Robustness to noise. Each function shows how the accuracy of a method at a certain bin of the occupancy histogram changes with increasing level of noise. See text for more details.

*Robustness to Noise*.  Figure 10 shows robustness of evaluated methods to noise. We randomly noisify the bunny input points with noise with normal distribution in {0,2,…, 10}*σ* surrounding ground input points. Next, for each noise level, we reconstruct the scene using a method and we compute the occupancy histogram of the reconstruction (see Section 9.1). We take a bin *B* from the occupancy histogram for each noise level and plot it as a piecewise linear occupancy function of the method at bin *B*. The occupancy function shows how the accuracy of a method at a certain bin of the occupancy histogram changes with the increasing level of noise. Figures 10(a), 10(b), 10(c), and 10(d) show the occupancy functions of Poisson, baseline, former, and proposed methods at bin 1 (2,3,10).

Figure 10(a) shows that the occupancy of Poisson and former methods at bin 1 decreases rapidly with increasing noise level while the occupancy of the baseline and the proposed methods at bin 1 decreases slowly and is almost at the same level.

Figures 10(b) and 10(c) show that the occupancy of Poisson and former methods at bins 2,3 increases slowly with increasing noise level while the occupancy of the baseline and the proposed methods at bins 2 and 3 increases rapidly almost identically.

Note that the more slowly the occupancy at bin 1 decreases and the more slowly the occupancy at bins ≥2 increases, the more accurate the reconstruction is.

Figure 10(d) shows that the occupancy of the Poisson method at bin 11 is much higher than all other methods. Note that bin 11 of the occupancy histogram is the number of pixels where acc(*x*, *c*) > 10; see Section 9.1.

We have experimentally shown that the proposed method is more accurate compared to the former method. Additionally, we showed that the proposed method produces the results at the same accuracy level as the baseline method and both the proposed and the baseline methods are much more accurate than the Poisson method with increasing the level of noise.

### 9.3. Accuracy Evaluation on Real-World Dataset

**Figure 11:**
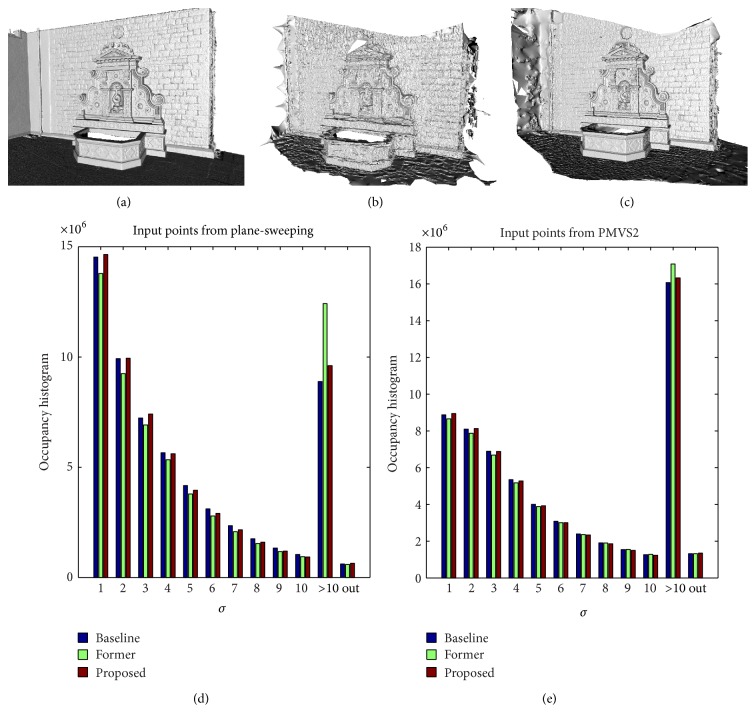
Accuracy evaluation on real-world dataset. (a) Ground truth generated by a laser scan, (b) reconstruction using the proposed method on input points generated by PMVS2 software, and (c) reconstruction using the proposed method on input points generated by the plane-sweeping approach described in [41]. (d) and (e) Bins 1 to 10 represent a histogram of reconstruction distances from ground-truth laser scan in units *σ*. Bin 11 is the number of values above 10*σ* and bin 12 is the number of wrong values. See text for more detailed description evaluation method. (d) Evaluations of reconstructions using baseline, former, and proposed method on input points generated by the plane-sweeping method. (e) Evaluations using input points generated by the PMVS2 software.

Figure 11 shows renderings and occupancy histograms of reconstructions using baseline, former, and proposed method with respect to a ground-truth laser scan. The laser scan, images, and calibrations were provided by Strecha et al. and downloaded from their evaluation page [6]. We have used two sources of point clouds as input to the reconstruction methods. The first one was generated by PMVS2 software (PMVS2 is software developed by Furukawa and Ponce [13]). The second one was generated by the plane-sweeping method described in [41].

Figures 11(a), 11(b), and 11(c) show the rendering of a ground-truth laser scan, the rendering of the reconstruction of proposed method on input points generated by PMVS2, and the rendering of reconstruction of proposed method on input points computed from the plane-sweeping method described in [41]. It shows that the proposed method works for input points generated by different MVS based methods.

Figure 11(d) shows the occupancy histograms of reconstructions of baseline, former, and proposed methods on [41] input points.  Figure 11(e) shows the occupancy histograms of reconstructions of baseline, former, and proposed methods on PMVS2 input points. Figures 11(d) and 11(e) show that baseline and proposed methods produce more accurate results than the former method. Additionally, it shows that the proposed method produces the results at the same accuracy level as the baseline method.

### 9.4. Evaluation on Middlebury Datasets

The datasets “templeRing” and “dinoRing” are provided for benchmarking multiview reconstruction methods [5] (see http://vision.middlebury.edu/mview/).

Figure 14 shows results of reconstruction of the “templeRing” and “dinoRing” datasets using the method proposed in this paper. Note that we have not used silhouette images at all.

Table 4 shows quantitative evaluation comparing our results with the laser-scanned ground truth. The accuracy values for the “templeRing” and “dinoRing” meshes are 0.53 mm and 0.4 mm, respectively. More remarkably, the completeness measures are 99.3% and 99.5%. The most relevant method evaluated in the Middlebury webpage is the method proposed in [35]. The method [35] first reconstructs the scene using the “baseline” method and then it refines the reconstruction using a final mesh refinement step. It is important to note that we do not use any final mesh refinement step in the proposed method. Nevertheless, the proposed method is among the best methods in the evaluation page. Of course, the results will vary if a different depth-map estimation method is employed.

### 9.5. Results of Proposed Method on Real-World Datasets

**Figure 12:**
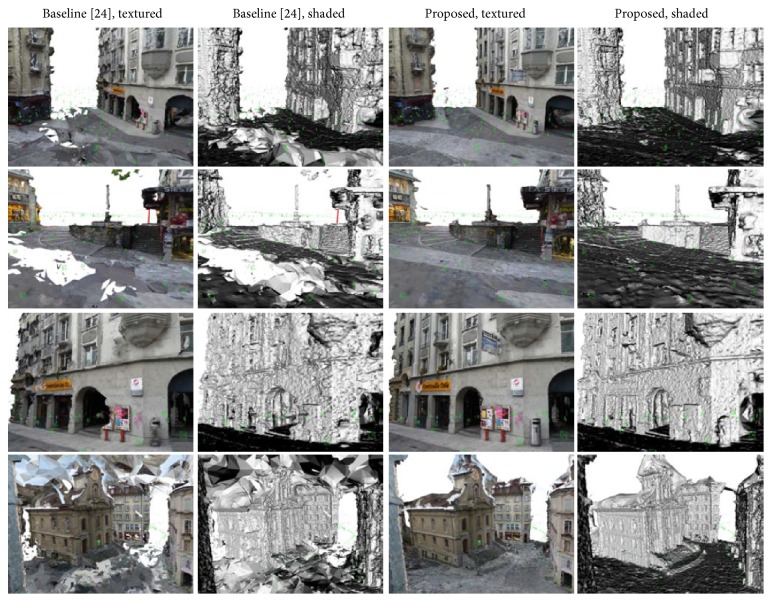
Results for the “Lausanne” dataset. Reconstruction using the baseline approach is in the first two columns. Reconstruction using our approach is in the second two columns. Textured results are shown in the first and the third columns of the images. Corresponding shaded results are in the second and the fourth columns. Weakly supported surfaces are better reconstructed using the proposed approach than by the baseline approach.

**Figure 13:**
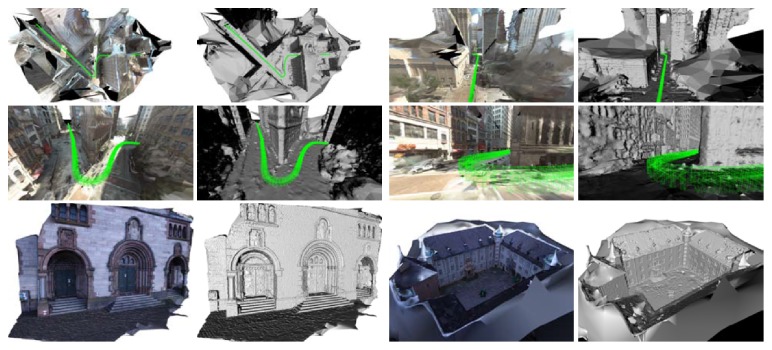
Results for the “googleStreetView,” “herzjesu,” and “castle” datasets. Odd columns textured, even shaded.

**Figure 14:**
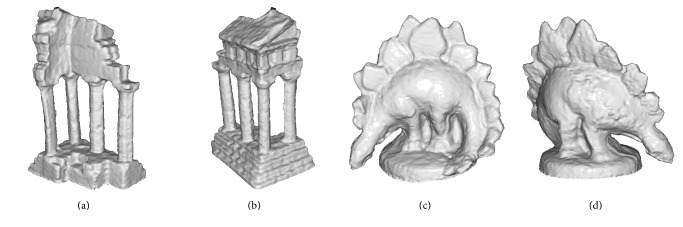
Middlebury results. Reconstruction results of Middlebury datasets “templeRing” and “dinoRing”.

Figures 13 (bottom row) and 12 demonstrate that the proposed method reconstructs weakly supported surfaces better than the baseline method. The weakly supported surface in Figures 13 and 12 is mostly the ground plane.  Figure 13 (first two rows) shows screen shots of other results using the proposed method where the weakly supported ground plane is reconstructed.

## 10. Conclusion and Future Work

We have presented a new surface reconstruction method that can reconstruct surfaces strongly supported by input points with the same accuracy as the state-of-the-art methods and moreover it can reconstruct weakly supported surfaces (e.g., low-textured walls, windows, cars, and ground planes). We assume calibrated sensors and input points augmented with visibility information of sensors on the input. We introduce an observation that it is also possible to reconstruct a surface that does not contain input points and we demonstrate it in an example. We assume an infinite number of ideal noiseless, outliers-less input points in the example. We accumulate free-space support from visibility information of input points and we show that nonzero free-space support is the evidence for free space and the zero free-space support is the evidence for full space or hallucination in the example. Therefore, the nonzero to zero change is the evidence of a surface (interface). Based on this observation, we define and study free-space support of tetrahedra in tetrahedralization of (real-world) input points that can be noisy and contain huge amount of outliers, and the number of input points is finite. We design an interface classifier based on the experiments. We experimentally show that the number of false positives (wrongly classified noninterface point as interface) is negligible and that the number of true positives (especially on weakly supported surfaces) is considerable. Finally, we propose a new method that extends an existing state-of-the-art (baseline) method by using an interface classifier, so that the existing state-of-the-art method gains the ability to reconstruct weakly supported surfaces. The newly proposed method strongly follows the existing (former) method that introduces the problem of weakly supported surfaces and is able to reconstruct them. However, we discuss and experimentally show that the newly proposed method produces more accurate results and reconstructs weakly supported surfaces better.

We introduce an observation that it is also possible to reconstruct an occluder surface that does not contain input points and we have proposed and evaluated methods that trade this observation in real-world scenario. However, to be practical, the method assumes that the occluder surface is weakly covered by the input points. A possible future work would be to try to use this observation in cases when the occluder is not covered by the input points at all. One of the possible ways would be to estimate possible parts of occluder presence in the space and introduce a new helper points into the tetrahedralization. It would be also possible to work with another volumetric representation of the space, that is, voxels, and so forth. Furthermore, we think that expanding our idea to the aerial reconstructions where facades are usually weakly supported would be nice direction of future work.

## Figures and Tables

**Algorithm 1:**
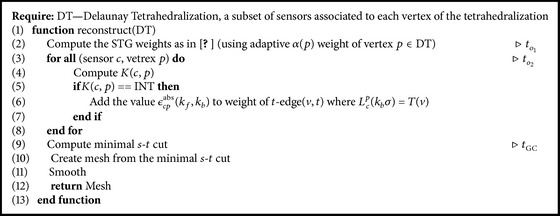
The algorithmic overview of the proposed method.

**Table 1:** Computed and used interface classifier *K* parameters.

	Parameter				
	*k*_abs_	*k*_*rel*⁡_	*k*_outl_	*k*_*f*_	*k*_*b*_
Estimated	0.3	500	700		
Used	0.1	1000	400	3	4

**Table 2:** Decision tables and classification results of interface classifier *K*(*c*, *p*) on different datasets.

	Interface	Noninterface
BunnySSS		
INT	82.7% TP	0.5% FP
NOI	17.3% FN	99.5% TN
BunnyWSS		
INT	41.7% TP	0.2% FP
NOI	58.3% FN	99.8% TN
BunnyWSSO		
INT	26.6% TP	0.2% FP
NOI	73.4% FN	99.8% TN
FountainSSS		
INT	55.4% TP	1.3% FP
NOI	44.6% FN	98.7% TN

**Table 3:** Performance data for different results.

Result name	*t*_*o*_1__	*t*_*o*_2__	*t*_*GC*_	*n*_*p*_	*T*_*t*_
Fountain	12:27	2:44	0:44	4.6 M	30.0 M
Street-view	05:00	2:34	0:52	2.9 M	19.6 M
Lausanne	20:02	3:28	0:53	4.9 M	32.7 M

*t*_*o*_1__ is the time of the first part of the proposed algorithm and *t*_*o*_2__ is the time of the second one. *t*_*GC*_ is the time of solving the minimal *s*-*t* cut problem. The times are in the format: minutes: seconds. *n*_*p*_ is the number of vertices and *T*_*t*_ is the number of tetrahedra in the tetrahedralization. The letter M stands for million.

**Table 4:** Middlebury results.

Method	“templeRing”		“dinoRing”	
	Acc.	Compl.	Acc.	Compl.
Hiep et al. [35]	0.45 mm	99.8%	0.53 mm	99.7%
Sinha et al. [32]	0.79 mm	94.9%	0.69 mm	97.2%
Proposed	0.53 mm	99.3%	0.4 mm	99.5%
